# Effects of the Trendelenburg Position and Positive End-Expiratory Pressure on the Internal Jugular Vein Cross-Sectional Area in Children With Simple Congenital Heart Defects

**DOI:** 10.1097/MD.0000000000003525

**Published:** 2016-05-06

**Authors:** Hee Yeong Kim, Jae Moon Choi, Yong-Hun Lee, Sukyung Lee, Hwanhee Yoo, Mijeung Gwak

**Affiliations:** From the Department of Anesthesiology and Pain Medicine (HYK), Hangang Sacred Heart Hospital, Hallym University College of Medicine; and Department of Anesthesiology and Pain Medicine (JMC, Y-HL, SL, HY, MG), Asan Medical Center, University of Ulsan College of Medicine, Seoul, Republic of Korea.

## Abstract

Catheterization of the internal jugular vein (IJV) remains difficult in pediatric populations. Increasing the cross-sectional area (CSA) of the IJV facilitates cannulation and decreases complications. We aimed to evaluate the Trendelenburg position and the levels of positive end-expiratory pressure (PEEP) at which the maximum increase of CSA of the IJV occurred in children undergoing cardiac surgery.

In this prospective study, the CSA of the right IJV was assessed using ultrasound in 47 anesthetized pediatric patients with simple congenital heart defects. The baseline CSA was obtained in response to a supine position with no PEEP and compared with 5 different randomly ordered maneuvers, that is, a PEEP of 5 and 10 cm H_2_O in a supine position and of 0, 5, and 10 cm H_2_O in a 10° Trendelenburg position. Hemodynamic variables, including blood pressure and heart rate, maximum and minimum diameters, and CSA, were measured.

All maneuvers increased the CSA of the right IJV with respect to the control condition. In the supine position, the CSA was increased by 9.4% with a PEEP of 5 and by 19.5% with a PEEP of 10 cm H_2_O. The Trendelenburg tilt alone increased the CSA by 19.0%, and combining the 10° Trendelenburg with a 10 cm H_2_O PEEP resulted in the largest IJV CSA increase (33.3%) compared with the supine position with no PEEP. Meanwhile, vital signs remained relatively steady during the experiment.

The application of the Trendelenburg position and a 10 cm H_2_O PEEP thus significantly increases the CSA of the right IJV, perhaps improving the chances of successful cannulation in pediatric patients with simple congenital heart defects.

## INTRODUCTION

Central venous catheterization is performed routinely to monitor central venous pressure and infuse vasoactive drugs in patients undergoing cardiac surgery. Catheterization of the right internal jugular vein (IJV) is usually preferred because the risk of pneumothorax is low, and the superior vena cava is nearly in a straight line with the IJV.^1^ However, percutaneous catheterization of the IJV remains difficult in pediatric patients and may be a special challenge even for the expert.

An ultrasound is helpful to localize the IJV and to define its size. The use of ultrasonography has been shown to improve cannulation success and to decrease the incidence of complications associated with catheter placement.^2,3^ Gordon et al reported that success on the first pass and reduction in complications were highly correlated with the diameter of the IJV.^4^

Several methods, including the Valsalva maneuver, Trendelenburg position, and positive end-expiratory pressure (PEEP), have been evaluated for increasing the cross-sectional area (CSA) of the IJV in adult patients.^5–7^ However, few studies have been performed to investigate how to increase the CSA of the IJV in children undergoing cardiac surgery. We thus aimed in our present study to evaluate using ultrasonography the Trendelenburg position and the levels of PEEP at which the maximum increase of CSA of the IJV occurred in children undergoing cardiac surgery.

## METHODS

This study was approved by the institutional review board of the Asan Medical Center (Reg. No.: 2015-0643) and was registered at cris.nih.go.kr (Reg. No.: KCT0001550). After obtaining signed informed consent from the parents, 47 children undergoing general anesthesia for elective simple congenital heart defect surgery were enrolled. Patients with a history of neck surgery, pulmonary disease, or previous right IJV cannulation were excluded. Patients with severe hypotension during the experiment were also excluded.

The children received intravenous premedication with 0.1 mg/kg midazolam. Routine monitoring of pulse oximetry, blood pressure, and an electrocardiogram were also performed. Anesthesia was induced with propofol, and rocuronium was used to facilitate the tracheal intubation. After endotracheal intubation, volume-controlled ventilation was initiated using a tidal volume of 8 mL/kg, inspiratory-expiratory ratio 1:2, an inspiratory oxygen fraction of 0.25, and no PEEP. The respiratory rate was adjusted to obtain normocapnia. Anesthesia was maintained with continuous infusions of propofol and remifentanil.

After anesthesia was induced, the radial artery was cannulated. Under continuous blood pressure monitoring, the patient was placed in a supine position, the head and neck were slightly extended using a roll placed under the shoulders, and the head was rotated 40°. Because the CSA of the right IJV varies in size depending on the mechanical ventilation, the ultrasonographic image was stored at end-inspiration when the largest area was shown. The baseline CSA was obtained in response to the supine position with no PEEP. The investigator was not blind to the maneuver applied. A L14–5sp linear array probe (Zonare Medical Systems, Mountain View, CA) was applied perpendicularly to the skin in a transverse orientation at the cricoid cartilage level. A single static image of the right IJV was obtained without pressure on the ultrasound probe by the same operator at each maneuver. The images were taken 1 min after applying the following maneuvers in random order: 5 and 10 cm H_2_O PEEP in a supine position and 0, 5, and 10 cm H_2_O PEEP in a 10° Trendelenburg position. The order in which maneuvers were applied to each patient was determined using the online GraphPad QuickCalcs randomization program (http://www.graphpad.com/quickcalcs/index.cfm). The hemodynamic variables, including blood pressure and heart rate, were monitored continuously during the study. The maximum and minimum diameters and CSA were measured using a program preloaded into Z.One Ultra (System Version 4.8.11C, Zonare Medical Systems) by a blind investigator.

The sample size was calculated based on 7 preliminary patients. The average difference in the CSA of the right IJV before and after applying a 10 cm H_2_O PEEP in 10° Trendelenburg was 9.9 mm^2^. A sample size of 43 patients was calculated in order to obtain 80% statistical power at a significance level of 0.05 (2-tailed). To allow for 10% loss during the study period, we intended to recruit a total of 47 patients. Continuous data were tested for a normal distribution using the Shapiro–Wilk test. Data were presented as mean ± standard deviation (SD) for normally distributed continuous variables and as numbers (%) for categorical variables. Continuous variables were compared using a repeated measures analysis of variance with Tukey's post-hoc comparison. A *P*-value < 0.05 was used as a threshold for statistically significant differences. Statistical analyses were performed using SPSS version 21.0 (IBM Corp., Armonk, NY).

## RESULTS

**FIGURE 1:**
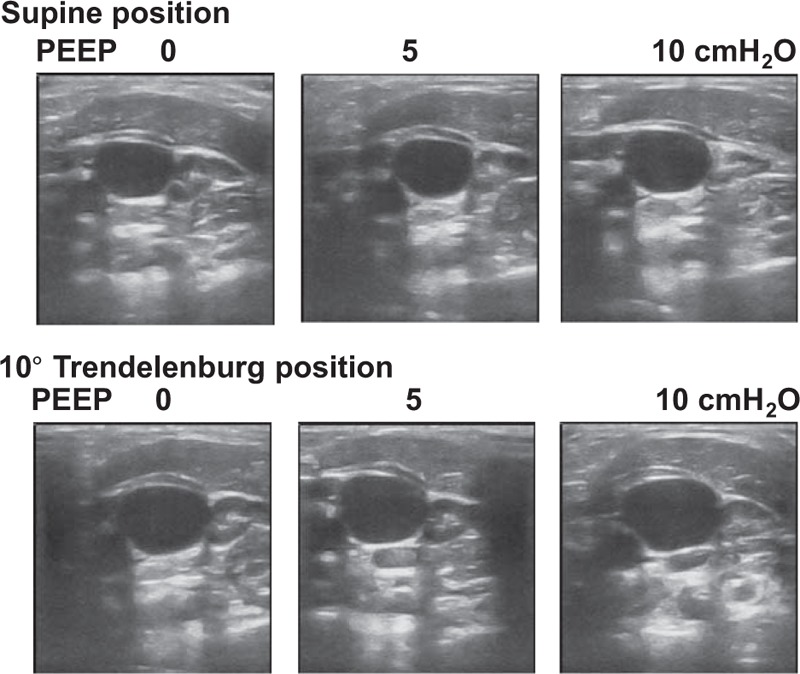
Ultrasound images of the right internal jugular vein.

None of the enrolled patients were excluded or withdrew from the study. There were no adverse events or complications related to the study protocol. The patient characteristics of the study subjects are presented in Table 1. A total of 282 measurements (6 maneuvers in 47 patients) were performed with the ultrasound procedure. Representative ultrasound images of the right IJV are shown in Figure 1.

**TABLE 1:**
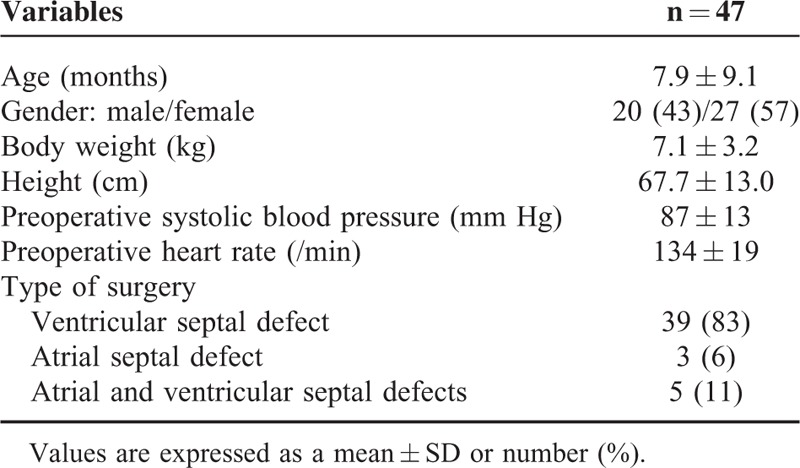
Patient Characteristics

**FIGURE 2:**
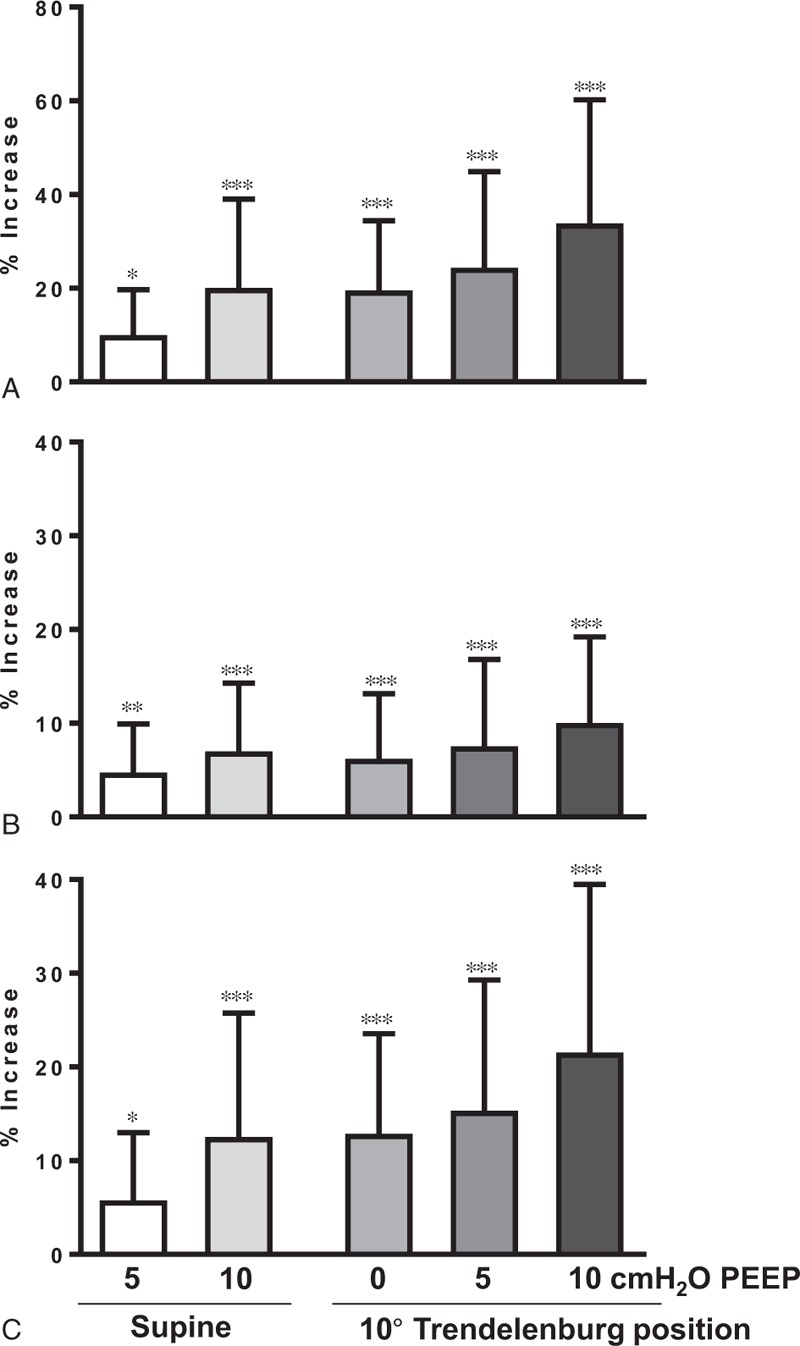
The mean increase in percentage of (A) the cross-sectional area, (B) the maximum diameter, and (C) the minimum diameter for each maneuver. Values are expressed as the mean ± SD. ^∗^*P* < 0.05, ^∗∗^*P* < 0.01, and ^∗∗∗^*P* < 0.001 versus supine position with no PEEP. PEEP = positive end-expiratory pressure.

Table 2 lists the vital sign changes and right IJV measurements in response to the maneuvers. The change in systolic blood pressure during a PEEP of 10 cm H_2_O in the supine position was statistically significant but not considered clinically relevant. All maneuvers significantly increased the CSA of the right IJV compared with the control condition. There were meaningful increases in the CSA as well as maximum and minimum diameters of the right IJV by raising the PEEP to 10 cm H_2_O. The CSA of the right IJV was similarly influenced by the maneuvers involving no PEEP in the 10° Trendelenburg position and with 10 cm H_2_O of PEEP in the supine position. The minimum diameters were increased more significantly than the maximum diameters with the application of PEEP and Trendelenburg maneuver. The mean increase in percentage of CSA, maximum, maximum diameter, and minimum diameter for each maneuver is presented in Figure 2. In the supine position, CSAs were increased by 9.4% (95% CI 6.4−12.4, *P* < 0.05) with a PEEP of 5 cm H_2_O and by 19.5% (95% CI 13.8−25.2, *P* < 0.001) with a PEEP of 10 cm H_2_O. The Trendelenburg tilt alone increased the CSA by 19.0% (95% CI 14.4−23.5, *P* < 0.001), and combining the 10° Trendelenburg position with 10 cm H_2_O of PEEP resulted in the largest increase in CSA of the IJV (33.3%, 95% CI 25.4–41.2, *P* < 0.001) as compared with the supine position with no PEEP.

**TABLE 2:**
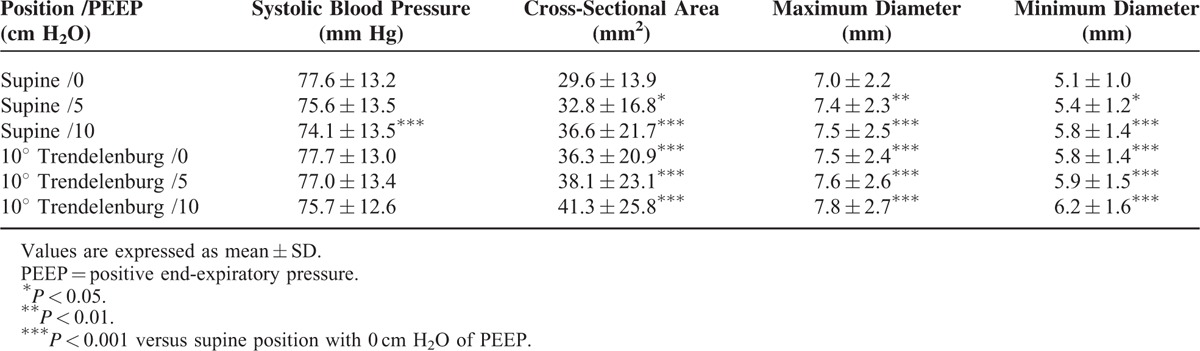
Vital Sign Changes and Right Internal Jugular Vein Measurements in Response to the Indicated Maneuvers

## DISCUSSION

We have investigated the Trendelenburg position and different levels of PEEP to increase the CSA of the right IJV. To our knowledge, our present study is the first to determine the response of the IJV in pediatric patients with a congenital heart defect to maneuvers to increase the CSA of the IJV. We found that both a 10° Trendelenburg position and a 10 cm H_2_O PEEP in the supine position were effective in increasing the CSA of the right IJV in children with simple congenital heart defects. Moreover, the CSA of the right IJV was increased by >30% in patients with a 10 cm H_2_O PEEP in 10° Trendelenburg. The maximum and minimum diameters were also significantly increased with the application of PEEP and Trendelenburg. Meanwhile, vital signs remained relatively steady during these procedures.

The placement of a central venous catheter is necessary in children undergoing cardiac surgery. The IJV is preferred to the subclavian vein for central venous cannulation because the latter is associated with a higher incidence of artery puncture.^8^ The right IJV is a commonly used route for access to the central vasculature because of its straight path into the right atrium. Using 2-dimensional ultrasound techniques is helpful to increase the success rates of catheter insertion.^9^ Considerable efforts have been directed toward increasing the CSA of the IJV as well as decreasing the complication rate during the procedure. Maneuvers that raise the intravascular pressure probably increase the CSA of the IJV. In adults, the use of Trendelenburg, ventilation with PEEP, a Valsalva maneuver, and hepatic compression significantly increases the CSA of the IJV.^6,7,10^

The Trendelenburg position decreases venous return from the IJV to the heart via the superior vena cava. In a prior study of the Trendelenburg effect on the IJV diameter, a 25° Trendelenburg position achieved the maximal distension. The authors of that study concluded that even a 10° tilt is effective in that greater angles of tilt are of little benefit and impractical in unstable patients.^11^ With a 10° Trendelenburg tilt, inspiratory hold of 20 cm H_2_O, and hepatic compression, the CSA of the IJV increased by 25%, 14%, and 26%, respectively, in adult patients.^10^ In addition, all interventions increased the intravascular pressure of the IJV significantly above control measurements. Increased intrathoracic pressure with PEEP distends the IJV by impeding venous return and compressing the superior vena cava.^12,13^ The CSAs of the IJV were also reported to increase by 16% and 22% during 5 and 10 cm H_2_O PEEP ventilation, respectively, in patients undergoing major cardiothoracic surgery.^6^ There were no further increases in CSA with greater PEEP >12 cm H_2_O.^7^

Several studies have been undertaken to increase the CSA of the IJV in pediatric patients. Botero and colleagues reported that the CSA of the right IJV is 36% greater than that of left IJV, and a positive inspiratory pressure hold significantly increased the CSA of the right IJV in healthy anesthetized children.^14^ Verghese and coworkers showed that a Valsalva maneuver with a positive inspiratory pressure of 25 mm Hg increased the right IJV size by 12.6% in infants in the supine position, and that a combination of the Valsalva, liver compression, and Trendelenburg offer the maximal increase in the CSA of the IJV.^15^ Placing patients in a 10° Trendelenburg position is common practice during cannulation of the IJV, and, as shown in our present study, can increase the CSA of the IJV by 19%. However, the effect of PEEP on the CSA of IJV has not yet been evaluated in pediatric patients. In the present study in children, we found that a 5 and 10 cm H_2_O PEEP increased the CSA of the IJV by 9% and 20%, respectively, in the supine position in accordance with the previous results in adults.^6^ Moreover, the CSA of the right IJV was increased by >30% in children in our study with a 10 cm H_2_O PEEP in the 10° Trendelenburg position. In our study series also, there was a significant difference in the CSA when comparing the Trendelenburg position with no PEEP to that with 10 cm H_2_O PEEP. This finding is in contrast to that of Marcus and colleagues, who reported that the CSA of the IJV in a 10° Trendelenburg positioned adult patient was not increased further by applying a 10 cm H_2_O PEEP.^6^

Some interindividual differences in the cross-sectional shape of the IJV were observed by ultrasonography in the present analyses. Most were elliptical, but a number of circles and tilted or distorted ellipses were also observed. Therefore, we measured the maximum and minimum diameters of the right IJV. In most cases, the former was the transverse diameter and the latter was the anteroposterior diameter. The degree of increase in the minimum diameter was higher than that of the maximum diameter (21.2% vs 9.7%) in patients with a 10 cm H_2_O PEEP in 10° Trendelenburg. These results may be of value in increasing the success rate and in decreasing the complication rate via preventing collapse of the IJV during cannulation. The increase in minimum diameter by 1.1 mm is worthwhile when considering the diameter of a 5 French catheter is 1.7 mm.

One notable limitation of our present study was that we did not determine the success rate and the extent to which central venous cannulation was improved. Further research should focus on the clinical usefulness of Trendelenburg and PEEP with respect to the success rate of central venous catheterization and patient outcomes. Another limitation of our present analysis was that the systolic blood pressure dropped slightly in the supine position with 10 cm H_2_O PEEP, which was statistically significant. However, it was not considered clinically significant as other vital signs were maintained stable. The PEEP maneuver is known to be a safe procedure in pediatric patients undergoing cardiac surgery for congenital heart disease as well as in children with acute respiratory failure. A previous study reported no significant changes in heart rate, mean arterial pressure, and right atrial pressure when 8 cm H_2_O PEEP was applied in pediatric patients undergoing cardiac surgery for congenital heart disease.^16^ Additionally, both the cardiac index and pulmonary compliance were well maintained at 12 cm H_2_O PEEP in infants and children with acute respiratory failure.^17^ Although the degree of blood pressure reduction with 10 cm H_2_O PEEP in the supine position was not considered clinically relevant in our study, it should be kept in mind that it does not imply that a high PEEP is safe in patients with complex congenital heart defects. An additional limitation of the present study was that ultrasound measurements are a user-dependent technology. Using a single ultrasonographer might have minimized interobserver variability in caliper placement.

In conclusion, both a 10° Trendelenburg tilt and a 10 cm H_2_O PEEP are effective maneuvers for increasing the CSA of the IJV, and a 10° Trendelenburg position in combination with 10 cm H_2_O of PEEP can additionally enhance this effect in children. The application of the Trendelenburg position and 10 cm H_2_O PEEP significantly increases the CSA of the right IJV, potentially improving the chances of a successful cannulation in pediatric patients.
